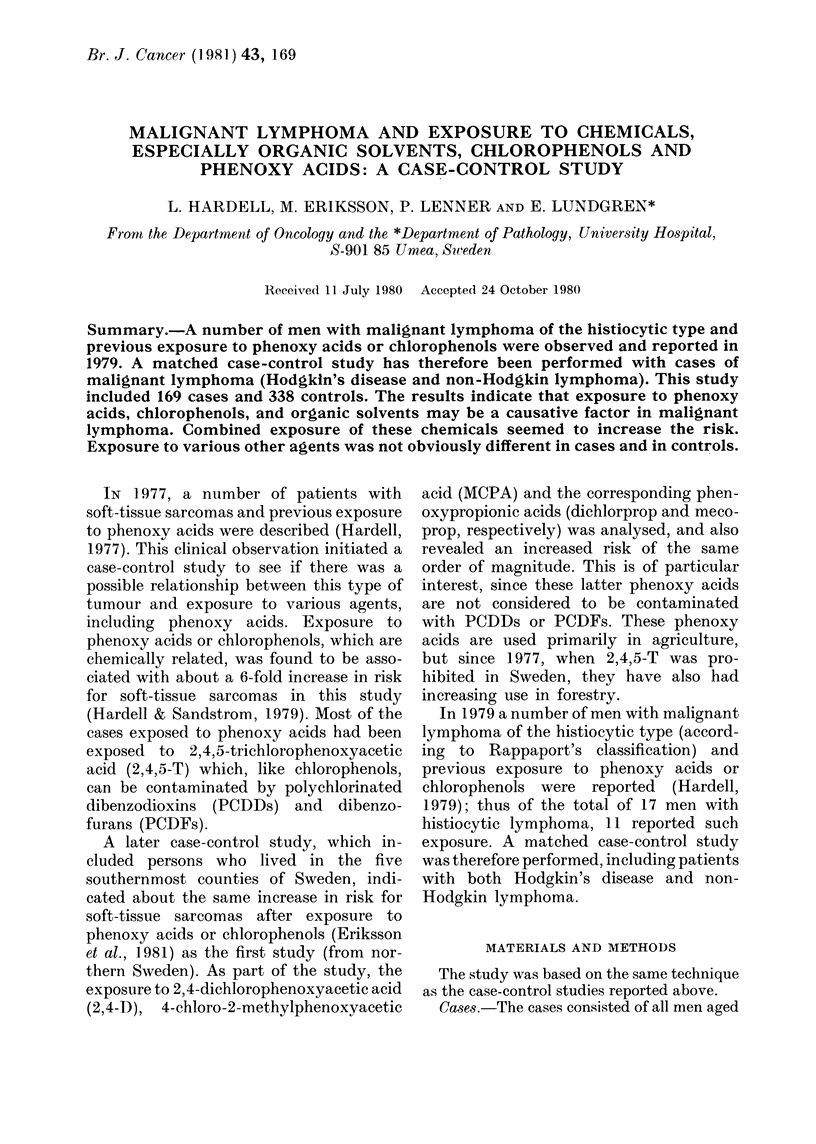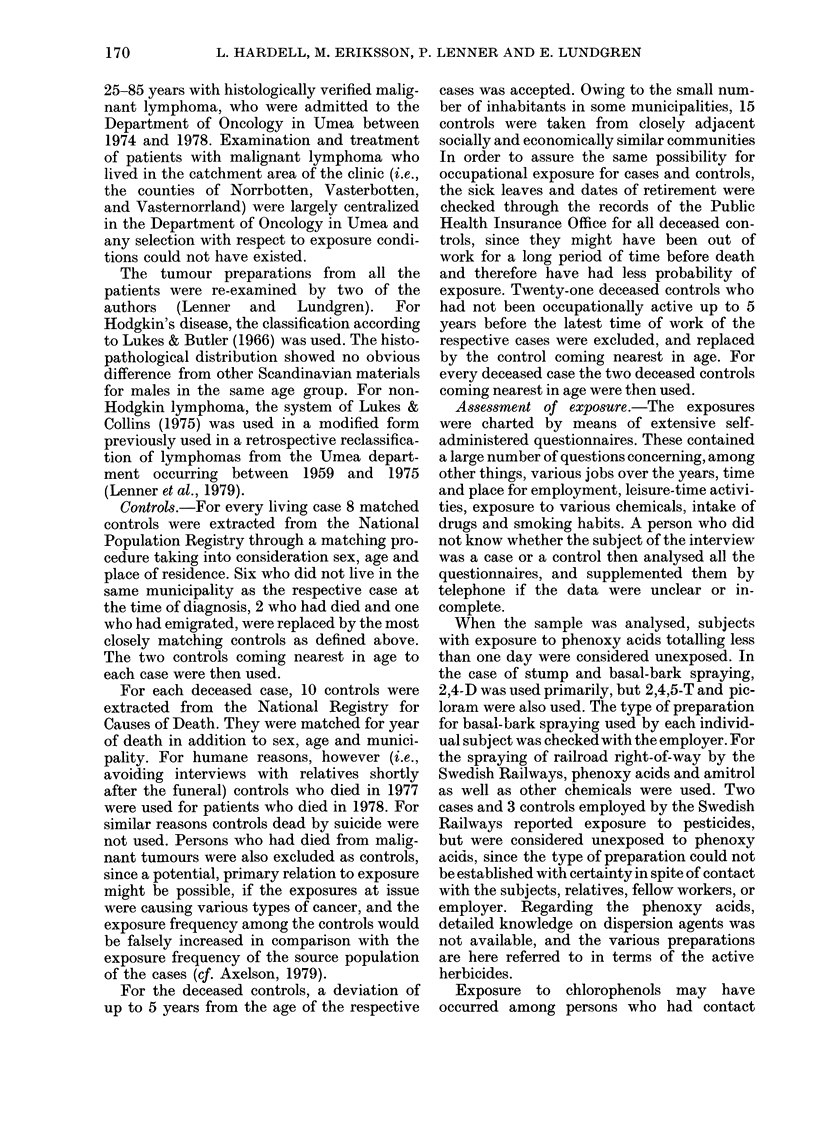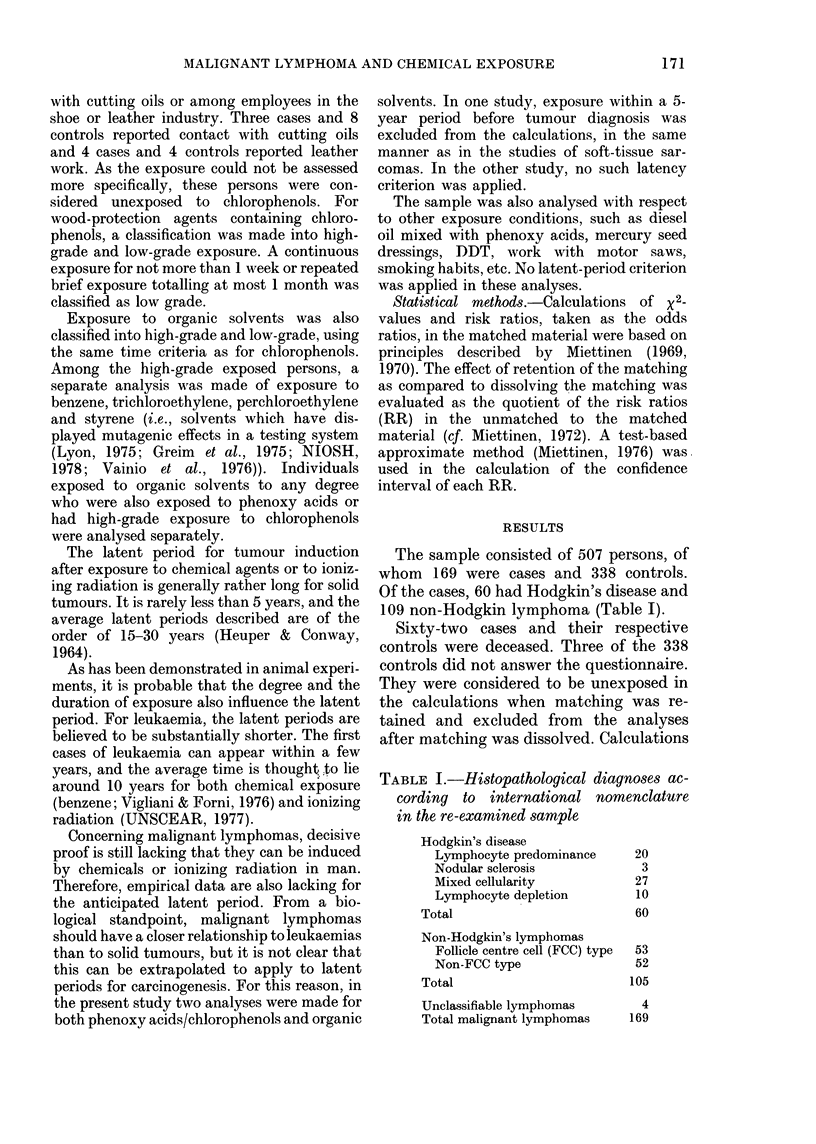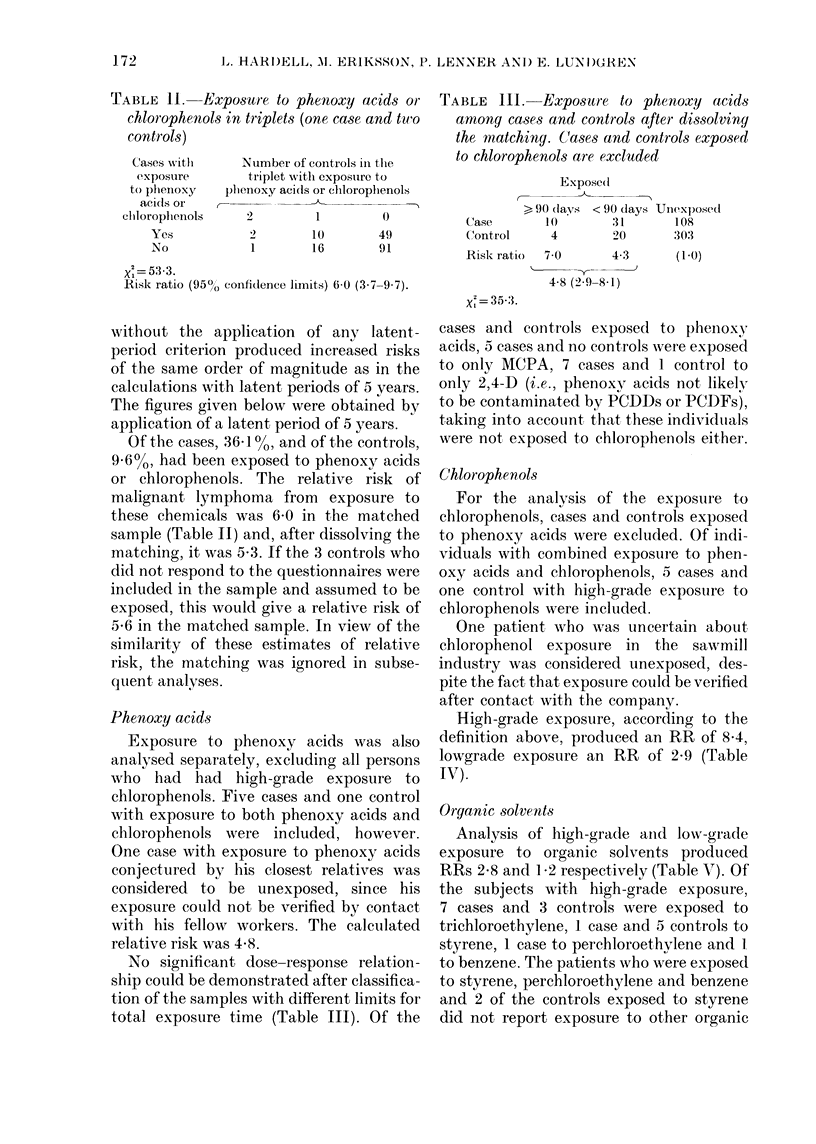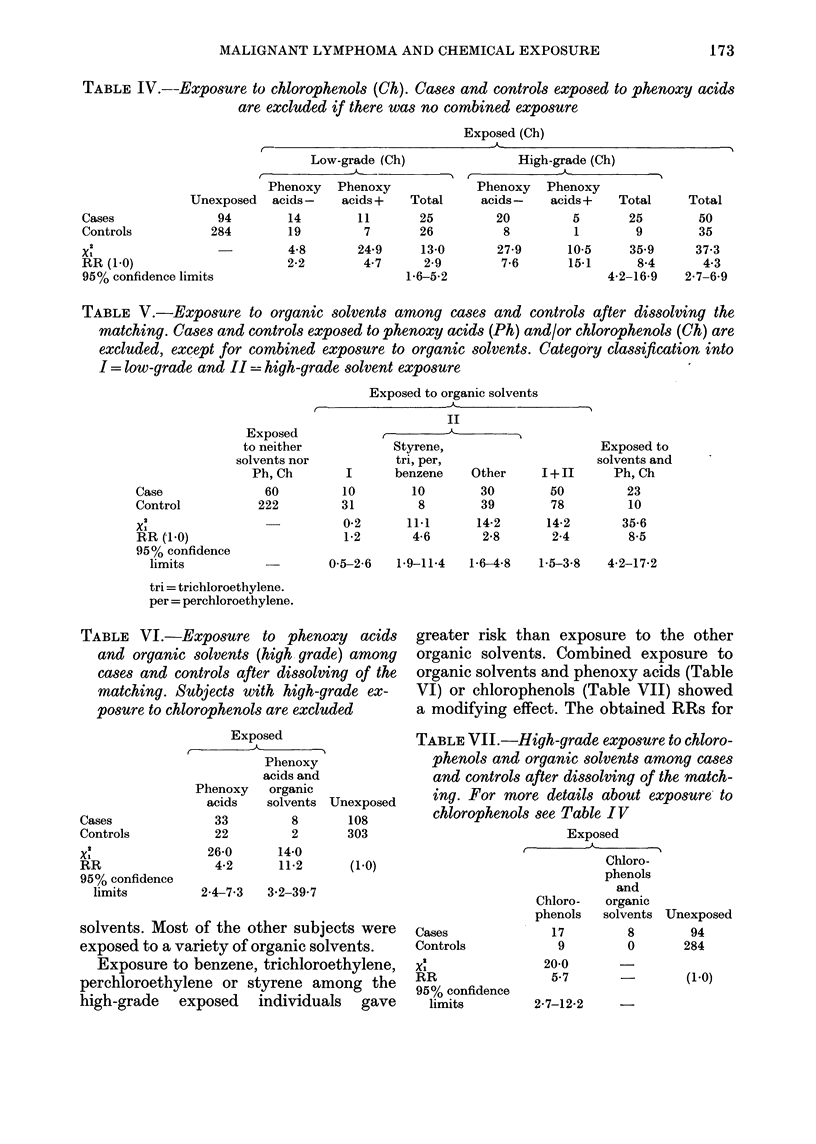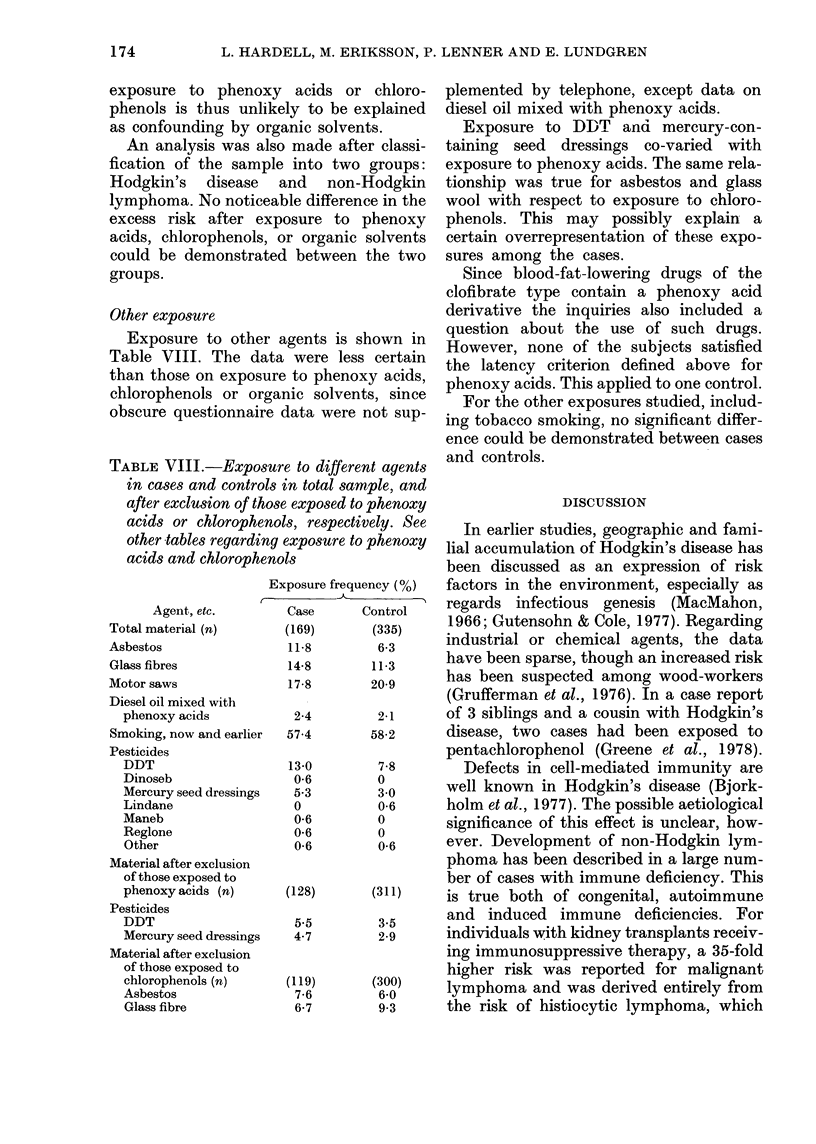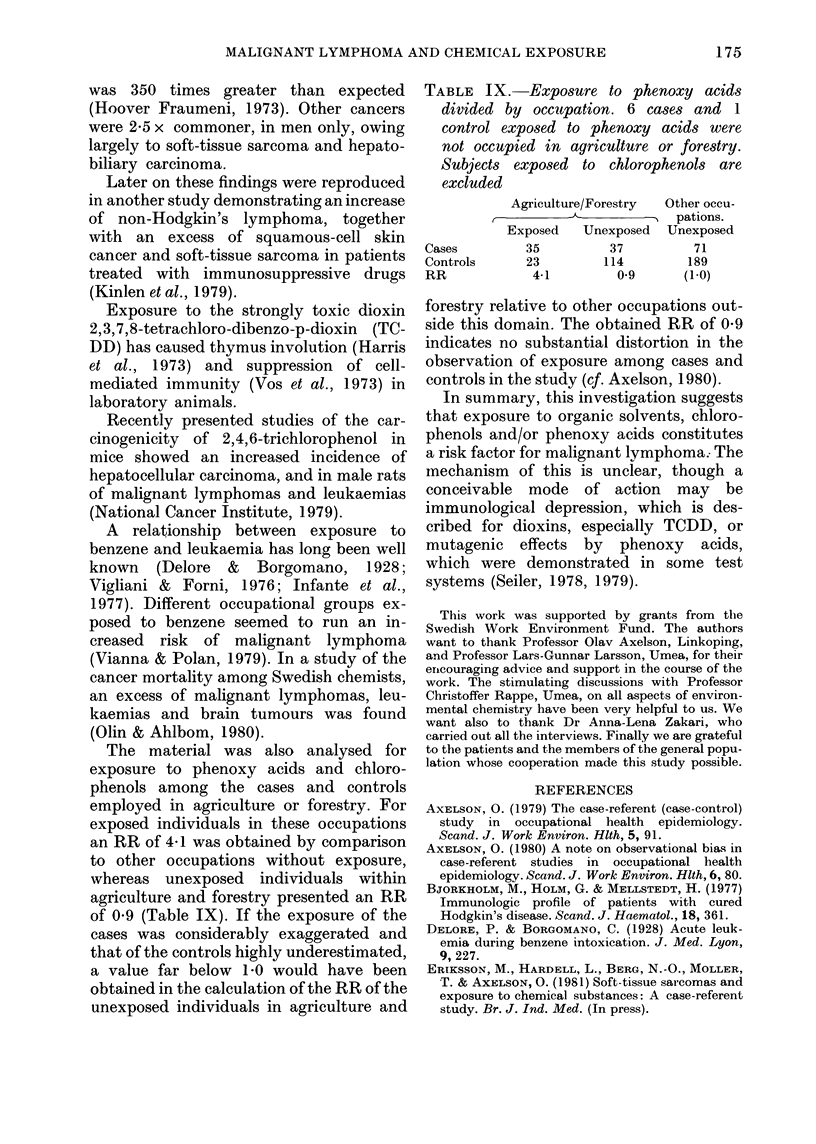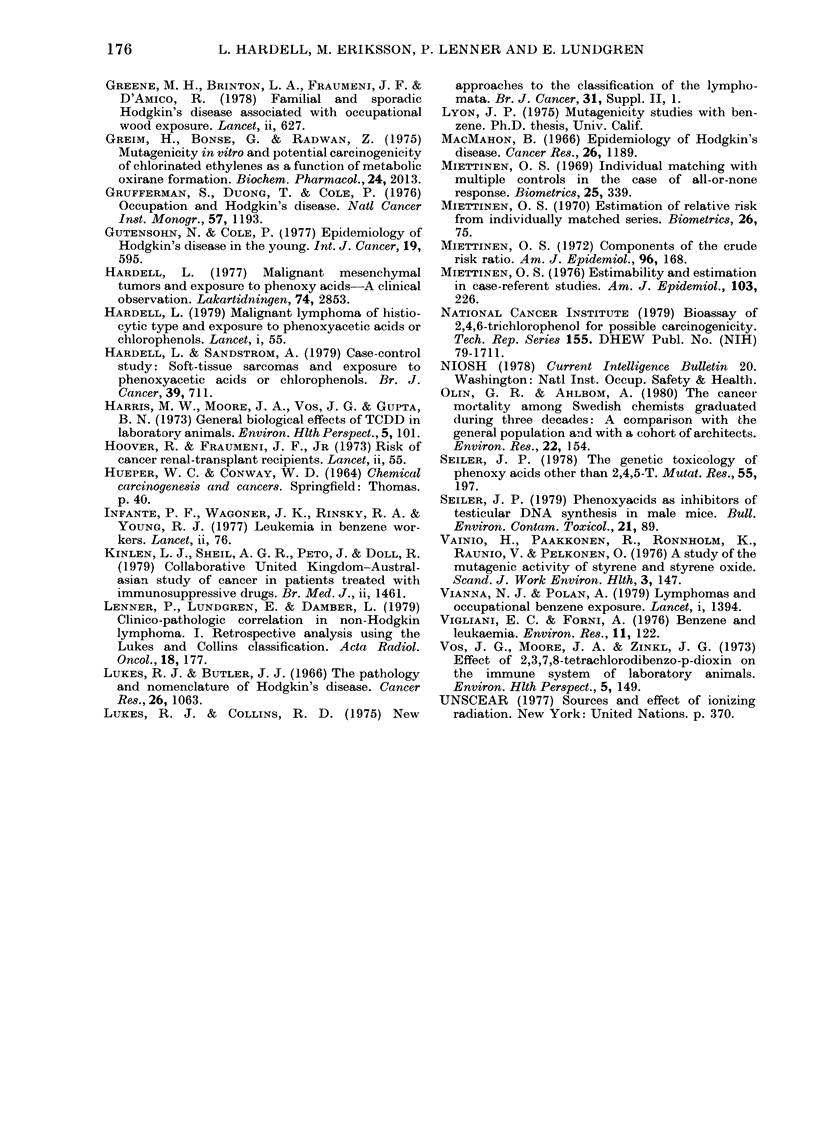# Malignant lymphoma and exposure to chemicals, especially organic solvents, chlorophenols and phenoxy acids: a case-control study.

**DOI:** 10.1038/bjc.1981.25

**Published:** 1981-02

**Authors:** L. Hardell, M. Eriksson, P. Lenner, E. Lundgren

## Abstract

A number of men with malignant lymphoma of the histiocytic type and previous exposure to phenoxy acids or chlorophenols were observed and reported in 1979. A matched case-control study has therefore been performed with cases of malignant lymphoma (Hodgkin's disease and non-Hodgkin lymphoma). This study included 169 cases and 338 controls. The results indicate that exposure to phenoxy acids, chlorophenols, and organic solvents may be a causative factor in malignant lymphoma. Combined exposure of these chemicals seemed to increase the risk. Exposure to various other agents was not obviously different in cases and in controls.


*Br. J. Cancer* (1981) **43**, 169

### MALIGNANT LYMPHOMA AND EXPOSURE TO CHEMICALS, ESPECIALLY ORGANIC SOLVENTS, CHLOROPHENOLS AND PHENOXY ACIDS: A CASE-CONTROL STUDY

L. HARDELL, M. ERIKSSON, P. LENNER AND E. LUNDGREN*

*From the Department of Oncology and the *Department of Pathology, University Hospital, S-901 85 Umea, Sweden*

Received 11 July 1980 Accepted 24 October 1980

**Summary.—A number of men with malignant lymphoma of the histiocytic type and previous exposure to phenoxy acids or chlorophenols were observed and reported in 1979. A matched case-control study has therefore been performed with cases of malignant lymphoma (Hodgkin's disease and non-Hodgkin lymphoma). This study included 169 cases and 338 controls. The results indicate that exposure to phenoxy acids, chlorophenols, and organic solvents may be a causative factor in malignant lymphoma. Combined exposure of these chemicals seemed to increase the risk. Exposure to various other agents was not obviously different in cases and in controls.**

In 1977, a number of patients with soft-tissue sarcomas and previous exposure to phenoxy acids were described (Hardell, 1977). This clinical observation initiated a case-control study to see if there was a possible relationship between this type of tumour and exposure to various agents, including phenoxy acids. Exposure to phenoxy acids or chlorophenols, which are chemically related, was found to be associated with about a 6-fold increase in risk for soft-tissue sarcomas in this study (Hardell & Sandstrom, 1979). Most of the cases exposed to phenoxy acids had been exposed to 2,4,5-trichlorophenoxyacetic acid (2,4,5-T) which, like chlorophenols, can be contaminated by polychlorinated dibenzodioxins (PCDDs) and dibenzofurans (PCDFs).

A later case-control study, which included persons who lived in the five southernmost counties of Sweden, indicated about the same increase in risk for soft-tissue sarcomas after exposure to phenoxy acids or chlorophenols (Eriksson *et al.*, 1981) as the first study (from northern Sweden). As part of the study, the exposure to 2,4-dichlorophenoxyacetic acid (2,4-D), 4-chloro-2-methylphenoxyacetic acid (MCPA) and the corresponding phenoxypropionic acids (dichlorprop and mecoprop, respectively) was analysed, and also revealed an increased risk of the same order of magnitude. This is of particular interest, since these latter phenoxy acids are not considered to be contaminated with PCDDs or PCDFs. These phenoxy acids are used primarily in agriculture, but since 1977, when 2,4,5-T was prohibited in Sweden, they have also had increasing use in forestry.

In 1979 a number of men with malignant lymphoma of the histiocytic type (according to Rappaport's classification) and previous exposure to phenoxy acids or chlorophenols were reported (Hardell, 1979); thus of the total of 17 men with histiocytic lymphoma, 11 reported such exposure. A matched case-control study was therefore performed, including patients with both Hodgkin's disease and non-Hodgkin lymphoma.

### MATERIALS AND METHODS

The study was based on the same technique as the case-control studies reported above.

*Cases.*—The cases consisted of all men aged

25–85 years with histologically verified malignant lymphoma, who were admitted to the Department of Oncology in Umea between 1974 and 1978. Examination and treatment of patients with malignant lymphoma who lived in the catchment area of the clinic (*i.e.*, the counties of Norrbotten, Vasterbotten, and Vasternorrland) were largely centralized in the Department of Oncology in Umea and any selection with respect to exposure conditions could not have existed.

The tumour preparations from all the patients were re-examined by two of the authors (Lenner and Lundgren). For Hodgkin's disease, the classification according to Lukes & Butler (1966) was used. The histopathological distribution showed no obvious difference from other Scandinavian materials for males in the same age group. For non-Hodgkin lymphoma, the system of Lukes & Collins (1975) was used in a modified form previously used in a retrospective reclassification of lymphomas from the Umea department occurring between 1959 and 1975 (Lenner *et al.*, 1979).

*Controls.*—For every living case 8 matched controls were extracted from the National Population Registry through a matching procedure taking into consideration sex, age and place of residence. Six who did not live in the same municipality as the respective case at the time of diagnosis, 2 who had died and one who had emigrated, were replaced by the most closely matching controls as defined above. The two controls coming nearest in age to each case were then used.

For each deceased case, 10 controls were extracted from the National Registry for Causes of Death. They were matched for year of death in addition to sex, age and municipality. For humane reasons, however (*i.e.*, avoiding interviews with relatives shortly after the funeral) controls who died in 1977 were used for patients who died in 1978. For similar reasons controls dead by suicide were not used. Persons who had died from malignant tumours were also excluded as controls, since a potential, primary relation to exposure might be possible, if the exposures at issue were causing various types of cancer, and the exposure frequency among the controls would be falsely increased in comparison with the exposure frequency of the source population of the cases (*cf.* Axelson, 1979).

For the deceased controls, a deviation of up to 5 years from the age of the respective cases was accepted. Owing to the small number of inhabitants in some municipalities, 15 controls were taken from closely adjacent socially and economically similar communities In order to assure the same possibility for occupational exposure for cases and controls, the sick leaves and dates of retirement were checked through the records of the Public Health Insurance Office for all deceased controls, since they might have been out of work for a long period of time before death and therefore have had less probability of exposure. Twenty-one deceased controls who had not been occupationally active up to 5 years before the latest time of work of the respective cases were excluded, and replaced by the control coming nearest in age. For every deceased case the two deceased controls coming nearest in age were then used.

*Assessment of exposure.*—The exposures were charted by means of extensive self-administered questionnaires. These contained a large number of questions concerning, among other things, various jobs over the years, time and place for employment, leisure-time activities, exposure to various chemicals, intake of drugs and smoking habits. A person who did not know whether the subject of the interview was a case or a control then analysed all the questionnaires, and supplemented them by telephone if the data were unclear or incomplete.

When the sample was analysed, subjects with exposure to phenoxy acids totalling less than one day were considered unexposed. In the case of stump and basal-bark spraying, 2,4-D was used primarily, but 2,4,5-T and picloram were also used. The type of preparation for basal-bark spraying used by each individual subject was checked with the employer. For the spraying of railroad right-of-way by the Swedish Railways, phenoxy acids and amitrol as well as other chemicals were used. Two cases and 3 controls employed by the Swedish Railways reported exposure to pesticides, but were considered unexposed to phenoxy acids, since the type of preparation could not be established with certainty in spite of contact with the subjects, relatives, fellow workers, or employer. Regarding the phenoxy acids, detailed knowledge on dispersion agents was not available, and the various preparations are here referred to in terms of the active herbicides.

Exposure to chlorophenols may have occurred among persons who had contact with cutting oils or among employees in the shoe or leather industry. Three cases and 8 controls reported contact with cutting oils and 4 cases and 4 controls reported leather work. As the exposure could not be assessed more specifically, these persons were considered unexposed to chlorophenols. For wood-protection agents containing chlorophenols, a classification was made into high-grade and low-grade exposure. A continuous exposure for not more than 1 week or repeated brief exposure totalling at most 1 month was classified as low grade.

Exposure to organic solvents was also classified into high-grade and low-grade, using the same time criteria as for chlorophenols. Among the high-grade exposed persons, a separate analysis was made of exposure to benzene, trichloroethylene, perchloroethylene and styrene (*i.e.*, solvents which have displayed mutagenic effects in a testing system (Lyon, 1975; Greim *et al.*, 1975; NIOSH, 1978; Vainio *et al.*, 1976)). Individuals exposed to organic solvents to any degree who were also exposed to phenoxy acids or had high-grade exposure to chlorophenols were analysed separately.

The latent period for tumour induction after exposure to chemical agents or to ionizing radiation is generally rather long for solid tumours. It is rarely less than 5 years, and the average latent periods described are of the order of 15–30 years (Heuper & Conway, 1964).

As has been demonstrated in animal experiments, it is probable that the degree and the duration of exposure also influence the latent period. For leukaemia, the latent periods are believed to be substantially shorter. The first cases of leukaemia can appear within a few years, and the average time is thought to lie around 10 years for both chemical exposure (benzene; Vigliani & Forni, 1976) and ionizing radiation (UNSCEAR, 1977).

Concerning malignant lymphomas, decisive proof is still lacking that they can be induced by chemicals or ionizing radiation in man. Therefore, empirical data are also lacking for the anticipated latent period. From a biological standpoint, malignant lymphomas should have a closer relationship to leukaemias than to solid tumours, but it is not clear that this can be extrapolated to apply to latent periods for carcinogenesis. For this reason, in the present study two analyses were made for both phenoxy acids/chlorophenols and organic solvents. In one study, exposure within a 5-year period before tumour diagnosis was excluded from the calculations, in the same manner as in the studies of soft-tissue sarcomas. In the other study, no such latency criterion was applied.

The sample was also analysed with respect to other exposure conditions, such as diesel oil mixed with phenoxy acids, mercury seed dressings, DDT, work with motor saws, smoking habits, etc. No latent-period criterion was applied in these analyses.

*Statistical methods.*—Calculations of $\chi^2$-values and risk ratios, taken as the odds ratios, in the matched material were based on principles described by Miettinen (1969, 1970). The effect of retention of the matching as compared to dissolving the matching was evaluated as the quotient of the risk ratios (RR) in the unmatched to the matched material (*cf.* Miettinen, 1972). A test-based approximate method (Miettinen, 1976) was used in the calculation of the confidence interval of each RR.

### RESULTS

The sample consisted of 507 persons, of whom 169 were cases and 338 controls. Of the cases, 60 had Hodgkin's disease and 109 non-Hodgkin lymphoma (Table I).

Sixty-two cases and their respective controls were deceased. Three of the 338 controls did not answer the questionnaire. They were considered to be unexposed in the calculations when matching was retained and excluded from the analyses after matching was dissolved. Calculations

TABLE I.—*Histopathological diagnoses according to international nomenclature in the re-examined sample*

| | |
|---|---|
| Hodgkin's disease | |
| Lymphocyte predominance | 20 |
| Nodular sclerosis | 3 |
| Mixed cellularity | 27 |
| Lymphocyte depletion | 10 |
| Total | 60 |
| Non-Hodgkin's lymphomas | |
| Follicle centre cell (FCC) type | 53 |
| Non-FCC type | 52 |
| Total | 105 |
| Unclassifiable lymphomas | 4 |
| Total malignant lymphomas | 169 |

TABLE II.—*Exposure to phenoxy acids or chlorophenols in triplets (one case and two controls)*

| Cases with exposure to phenoxy acids or chlorophenols | Number of controls in the triplet with exposure to phenoxy acids or chlorophenols | | |
|---|---|---|---|
| | 2 | 1 | 0 |
| Yes | 2 | 10 | 49 |
| No | 1 | 16 | 91 |

$\chi_1^2 = 53 \cdot 3$.

Risk ratio (95% confidence limits) 6·0 (3·7–9·7).

TABLE III.—*Exposure to phenoxy acids among cases and controls after dissolving the matching. Cases and controls exposed to chlorophenols are excluded*

| | Exposed | | |
|---|---|---|---|
| | ≥ 90 days | < 90 days | Unexposed |
| Case | 10 | 31 | 108 |
| Control | 4 | 20 | 303 |
| Risk ratio | 7·0 | 4·3 | (1·0) |
| | 4·8 (2·9–8·1) | | |

$\chi_1^2 = 35 \cdot 3$.

without the application of any latent-period criterion produced increased risks of the same order of magnitude as in the calculations with latent periods of 5 years. The figures given below were obtained by application of a latent period of 5 years.

Of the cases, 36·1%, and of the controls, 9·6%, had been exposed to phenoxy acids or chlorophenols. The relative risk of malignant lymphoma from exposure to these chemicals was 6·0 in the matched sample (Table II) and, after dissolving the matching, it was 5·3. If the 3 controls who did not respond to the questionnaires were included in the sample and assumed to be exposed, this would give a relative risk of 5·6 in the matched sample. In view of the similarity of these estimates of relative risk, the matching was ignored in subsequent analyses.

*Phenoxy acids*

Exposure to phenoxy acids was also analysed separately, excluding all persons who had had high-grade exposure to chlorophenols. Five cases and one control with exposure to both phenoxy acids and chlorophenols were included, however. One case with exposure to phenoxy acids conjectured by his closest relatives was considered to be unexposed, since his exposure could not be verified by contact with his fellow workers. The calculated relative risk was 4·8.

No significant dose–response relationship could be demonstrated after classification of the samples with different limits for total exposure time (Table III). Of the cases and controls exposed to phenoxy acids, 5 cases and no controls were exposed to only MCPA, 7 cases and 1 control to only 2,4-D (*i.e.*, phenoxy acids not likely to be contaminated by PCDDs or PCDFs), taking into account that these individuals were not exposed to chlorophenols either.

*Chlorophenols*

For the analysis of the exposure to chlorophenols, cases and controls exposed to phenoxy acids were excluded. Of individuals with combined exposure to phenoxy acids and chlorophenols, 5 cases and one control with high-grade exposure to chlorophenols were included.

One patient who was uncertain about chlorophenol exposure in the sawmill industry was considered unexposed, despite the fact that exposure could be verified after contact with the company.

High-grade exposure, according to the definition above, produced an RR of 8·4, lowgrade exposure an RR of 2·9 (Table IV).

*Organic solvents*

Analysis of high-grade and low-grade exposure to organic solvents produced RRs 2·8 and 1·2 respectively (Table V). Of the subjects with high-grade exposure, 7 cases and 3 controls were exposed to trichloroethylene, 1 case and 5 controls to styrene, 1 case to perchloroethylene and 1 to benzene. The patients who were exposed to styrene, perchloroethylene and benzene and 2 of the controls exposed to styrene did not report exposure to other organic

Table IV.—*Exposure to chlorophenols (Ch). Cases and controls exposed to phenoxy acids are excluded if there was no combined exposure*

| | | Exposed (Ch) | | | | | | |
|---|---|---|---|---|---|---|---|---|
| | | Low-grade (Ch) | | | High-grade (Ch) | | | |
| | Unexposed | Phenoxy acids − | Phenoxy acids + | Total | Phenoxy acids − | Phenoxy acids + | Total | Total |
| Cases | 94 | 14 | 11 | 25 | 20 | 5 | 25 | 50 |
| Controls | 284 | 19 | 7 | 26 | 8 | 1 | 9 | 35 |
| $\chi_1^2$ | — | 4·8 | 24·9 | 13·0 | 27·9 | 10·5 | 35·9 | 37·3 |
| RR (1·0) | | 2·2 | 4·7 | 2·9 | 7·6 | 15·1 | 8·4 | 4·3 |
| 95% confidence limits | | | | 1·6–5·2 | | | 4·2–16·9 | 2·7–6·9 |

Table V.—*Exposure to organic solvents among cases and controls after dissolving the matching. Cases and controls exposed to phenoxy acids (Ph) and/or chlorophenols (Ch) are excluded, except for combined exposure to organic solvents. Category classification into I = low-grade and II = high-grade solvent exposure*

| | | Exposed to organic solvents | | | | |
|---|---|---|---|---|---|---|
| | | | II | | | |
| | Exposed to neither solvents nor Ph, Ch | I | Styrene, tri, per, benzene | Other | I+II | Exposed to solvents and Ph, Ch |
| Case | 60 | 10 | 10 | 30 | 50 | 23 |
| Control | 222 | 31 | 8 | 39 | 78 | 10 |
| $\chi_1^2$ | — | 0·2 | 11·1 | 14·2 | 14·2 | 35·6 |
| RR (1·0) | | 1·2 | 4·6 | 2·8 | 2·4 | 8·5 |
| 95% confidence limits | — | 0·5–2·6 | 1·9–11·4 | 1·6–4·8 | 1·5–3·8 | 4·2–17·2 |

tri = trichloroethylene.
per = perchloroethylene.

Table VI.—*Exposure to phenoxy acids and organic solvents (high grade) among cases and controls after dissolving of the matching. Subjects with high-grade exposure to chlorophenols are excluded*

| | Exposed | | |
|---|---|---|---|
| | Phenoxy acids | Phenoxy acids and organic solvents | Unexposed |
| Cases | 33 | 8 | 108 |
| Controls | 22 | 2 | 303 |
| $\chi_1^2$ | 26·0 | 14·0 | |
| RR | 4·2 | 11·2 | (1·0) |
| 95% confidence limits | 2·4–7·3 | 3·2–39·7 | |

solvents. Most of the other subjects were exposed to a variety of organic solvents.

Exposure to benzene, trichloroethylene, perchloroethylene or styrene among the high-grade exposed individuals gave greater risk than exposure to the other organic solvents. Combined exposure to organic solvents and phenoxy acids (Table VI) or chlorophenols (Table VII) showed a modifying effect. The obtained RRs for

Table VII.—*High-grade exposure to chlorophenols and organic solvents among cases and controls after dissolving of the matching. For more details about exposure to chlorophenols see Table IV*

| | Exposed | | |
|---|---|---|---|
| | Chlorophenols | Chlorophenols and organic solvents | Unexposed |
| Cases | 17 | 8 | 94 |
| Controls | 9 | 0 | 284 |
| $\chi_1^2$ | 20·0 | — | |
| RR | 5·7 | — | (1·0) |
| 95% confidence limits | 2·7–12·2 | — | |

exposure to phenoxy acids or chlorophenols is thus unlikely to be explained as confounding by organic solvents.

An analysis was also made after classification of the sample into two groups: Hodgkin's disease and non-Hodgkin lymphoma. No noticeable difference in the excess risk after exposure to phenoxy acids, chlorophenols, or organic solvents could be demonstrated between the two groups.

### *Other exposure*

Exposure to other agents is shown in Table VIII. The data were less certain than those on exposure to phenoxy acids, chlorophenols or organic solvents, since obscure questionnaire data were not supplemented by telephone, except data on diesel oil mixed with phenoxy acids.

Table VIII.—*Exposure to different agents in cases and controls in total sample, and after exclusion of those exposed to phenoxy acids or chlorophenols, respectively. See other tables regarding exposure to phenoxy acids and chlorophenols*

| | Exposure frequency (%) | |
|---|---|---|
| Agent, *etc.* | Case | Control |
| Total material (*n*) | (169) | (335) |
| Asbestos | 11·8 | 6·3 |
| Glass fibres | 14·8 | 11·3 |
| Motor saws | 17·8 | 20·9 |
| Diesel oil mixed with phenoxy acids | 2·4 | 2·1 |
| Smoking, now and earlier | 57·4 | 58·2 |
| Pesticides | | |
| DDT | 13·0 | 7·8 |
| Dinoseb | 0·6 | 0 |
| Mercury seed dressings | 5·3 | 3·0 |
| Lindane | 0 | 0·6 |
| Maneb | 0·6 | 0 |
| Reglone | 0·6 | 0 |
| Other | 0·6 | 0·6 |
| Material after exclusion of those exposed to phenoxy acids (*n*) | (128) | (311) |
| Pesticides | | |
| DDT | 5·5 | 3·5 |
| Mercury seed dressings | 4·7 | 2·9 |
| Material after exclusion of those exposed to chlorophenols (*n*) | (119) | (300) |
| Asbestos | 7·6 | 6·0 |
| Glass fibre | 6·7 | 9·3 |

Exposure to DDT and mercury-containing seed dressings co-varied with exposure to phenoxy acids. The same relationship was true for asbestos and glass wool with respect to exposure to chlorophenols. This may possibly explain a certain overrepresentation of these exposures among the cases.

Since blood-fat-lowering drugs of the clofibrate type contain a phenoxy acid derivative the inquiries also included a question about the use of such drugs. However, none of the subjects satisfied the latency criterion defined above for phenoxy acids. This applied to one control.

For the other exposures studied, including tobacco smoking, no significant difference could be demonstrated between cases and controls.

### DISCUSSION

In earlier studies, geographic and familial accumulation of Hodgkin's disease has been discussed as an expression of risk factors in the environment, especially as regards infectious genesis (MacMahon, 1966; Gutensohn & Cole, 1977). Regarding industrial or chemical agents, the data have been sparse, though an increased risk has been suspected among wood-workers (Grufferman *et al.*, 1976). In a case report of 3 siblings and a cousin with Hodgkin's disease, two cases had been exposed to pentachlorophenol (Greene *et al.*, 1978).

Defects in cell-mediated immunity are well known in Hodgkin's disease (Bjorkholm *et al.*, 1977). The possible aetiological significance of this effect is unclear, however. Development of non-Hodgkin lymphoma has been described in a large number of cases with immune deficiency. This is true both of congenital, autoimmune and induced immune deficiencies. For individuals with kidney transplants receiving immunosuppressive therapy, a 35-fold higher risk was reported for malignant lymphoma and was derived entirely from the risk of histiocytic lymphoma, which was 350 times greater than expected (Hoover Fraumeni, 1973). Other cancers were 2·5 × commoner, in men only, owing largely to soft-tissue sarcoma and hepatobiliary carcinoma.

Later on these findings were reproduced in another study demonstrating an increase of non-Hodgkin's lymphoma, together with an excess of squamous-cell skin cancer and soft-tissue sarcoma in patients treated with immunosuppressive drugs (Kinlen *et al.*, 1979).

Exposure to the strongly toxic dioxin 2,3,7,8-tetrachloro-dibenzo-p-dioxin (TCDD) has caused thymus involution (Harris *et al.*, 1973) and suppression of cell-mediated immunity (Vos *et al.*, 1973) in laboratory animals.

Recently presented studies of the carcinogenicity of 2,4,6-trichlorophenol in mice showed an increased incidence of hepatocellular carcinoma, and in male rats of malignant lymphomas and leukaemias (National Cancer Institute, 1979).

A relationship between exposure to benzene and leukaemia has long been well known (Delore & Borgomano, 1928; Vigliani & Forni, 1976; Infante *et al.*, 1977). Different occupational groups exposed to benzene seemed to run an increased risk of malignant lymphoma (Vianna & Polan, 1979). In a study of the cancer mortality among Swedish chemists, an excess of malignant lymphomas, leukaemias and brain tumours was found (Olin & Ahlbom, 1980).

The material was also analysed for exposure to phenoxy acids and chlorophenols among the cases and controls employed in agriculture or forestry. For exposed individuals in these occupations an RR of 4·1 was obtained by comparison to other occupations without exposure, whereas unexposed individuals within agriculture and forestry presented an RR of 0·9 (Table IX). If the exposure of the cases was considerably exaggerated and that of the controls highly underestimated, a value far below 1·0 would have been obtained in the calculation of the RR of the unexposed individuals in agriculture and forestry relative to other occupations outside this domain. The obtained RR of 0·9 indicates no substantial distortion in the observation of exposure among cases and controls in the study (*cf.* Axelson, 1980).

TABLE IX.—*Exposure to phenoxy acids divided by occupation. 6 cases and 1 control exposed to phenoxy acids were not occupied in agriculture or forestry. Subjects exposed to chlorophenols are excluded*

| | Agriculture/Forestry | | Other occupations. |
|---|---|---|---|
| | Exposed | Unexposed | Unexposed |
| Cases | 35 | 37 | 71 |
| Controls | 23 | 114 | 189 |
| RR | 4·1 | 0·9 | (1·0) |

In summary, this investigation suggests that exposure to organic solvents, chlorophenols and/or phenoxy acids constitutes a risk factor for malignant lymphoma. The mechanism of this is unclear, though a conceivable mode of action may be immunological depression, which is described for dioxins, especially TCDD, or mutagenic effects by phenoxy acids, which were demonstrated in some test systems (Seiler, 1978, 1979).

This work was supported by grants from the Swedish Work Environment Fund. The authors want to thank Professor Olav Axelson, Linkoping, and Professor Lars-Gunnar Larsson, Umea, for their encouraging advice and support in the course of the work. The stimulating discussions with Professor Christoffer Rappe, Umea, on all aspects of environmental chemistry have been very helpful to us. We want also to thank Dr Anna-Lena Zakari, who carried out all the interviews. Finally we are grateful to the patients and the members of the general population whose cooperation made this study possible.

### REFERENCES

AXELSON, O. (1979) The case-referent (case-control) study in occupational health epidemiology. *Scand. J. Work Environ. Hlth*, **5**, 91.

AXELSON, O. (1980) A note on observational bias in case-referent studies in occupational health epidemiology. *Scand. J. Work Environ. Hlth*, **6**, 80.

BJORKHOLM, M., HOLM, G. & MELLSTEDT, H. (1977) Immunologic profile of patients with cured Hodgkin's disease. *Scand. J. Haematol.*, **18**, 361.

DELORE, P. & BORGOMANO, C. (1928) Acute leukemia during benzene intoxication. *J. Med. Lyon*, **9**, 227.

ERIKSSON, M., HARDELL, L., BERG, N.-O., MOLLER, T. & AXELSON, O. (1981) Soft-tissue sarcomas and exposure to chemical substances: A case-referent study. *Br. J. Ind. Med.* (In press).

GREENE, M. H., BRINTON, L. A., FRAUMENI, J. F. & D'AMICO, R. (1978) Familial and sporadic Hodgkin's disease associated with occupational wood exposure. *Lancet*, ii, 627.

GREIM, H., BONSE, G. & RADWAN, Z. (1975) Mutagenicity *in vitro* and potential carcinogenicity of chlorinated ethylenes as a function of metabolic oxirane formation. *Biochem. Pharmacol.*, **24**, 2013.

GRUFFERMAN, S., DUONG, T. & COLE, P. (1976) Occupation and Hodgkin's disease. *Natl Cancer Inst. Monogr.*, **57**, 1193.

GUTENSOHN, N. & COLE, P. (1977) Epidemiology of Hodgkin's disease in the young. *Int. J. Cancer*, **19**, 595.

HARDELL, L. (1977) Malignant mesenchymal tumors and exposure to phenoxy acids—A clinical observation. *Lakartidningen*, **74**, 2853.

HARDELL, L. (1979) Malignant lymphoma of histiocytic type and exposure to phenoxyacetic acids or chlorophenols. *Lancet*, i, 55.

HARDELL, L. & SANDSTROM, A. (1979) Case-control study: Soft-tissue sarcomas and exposure to phenoxyacetic acids or chlorophenols. *Br. J. Cancer*, **39**, 711.

HARRIS, M. W., MOORE, J. A., VOS, J. G. & GUPTA, B. N. (1973) General biological effects of TCDD in laboratory animals. *Environ. Hlth Perspect.*, **5**, 101.

HOOVER, R. & FRAUMENI, J. F., JR (1973) Risk of cancer renal-transplant recipients. *Lancet*, ii, 55.

HUEPER, W. C. & CONWAY, W. D. (1964) *Chemical carcinogenesis and cancers*. Springfield: Thomas. p. 40.

INFANTE, P. F., WAGONER, J. K., RINSKY, R. A. & YOUNG, R. J. (1977) Leukemia in benzene workers. *Lancet*, ii, 76.

KINLEN, L. J., SHEIL, A. G. R., PETO, J. & DOLL, R. (1979) Collaborative United Kingdom–Australasian study of cancer in patients treated with immunosuppressive drugs. *Br. Med. J.*, ii, 1461.

LENNER, P., LUNDGREN, E. & DAMBER, L. (1979) Clinico-pathologic correlation in non-Hodgkin lymphoma. I. Retrospective analysis using the Lukes and Collins classification. *Acta Radiol. Oncol.*, **18**, 177.

LUKES, R. J. & BUTLER, J. J. (1966) The pathology and nomenclature of Hodgkin's disease. *Cancer Res.*, **26**, 1063.

LUKES, R. J. & COLLINS, R. D. (1975) New approaches to the classification of the lymphomata. *Br. J. Cancer*, **31**, Suppl. II, 1.

LYON, J. P. (1975) Mutagenicity studies with benzene. Ph.D. thesis, Univ. Calif.

MACMAHON, B. (1966) Epidemiology of Hodgkin's disease. *Cancer Res.*, **26**, 1189.

MIETTINEN, O. S. (1969) Individual matching with multiple controls in the case of all-or-none response. *Biometrics*, **25**, 339.

MIETTINEN, O. S. (1970) Estimation of relative risk from individually matched series. *Biometrics*, **26**, 75.

MIETTINEN, O. S. (1972) Components of the crude risk ratio. *Am. J. Epidemiol.*, **96**, 168.

MIETTINEN, O. S. (1976) Estimability and estimation in case-referent studies. *Am. J. Epidemiol.*, **103**, 226.

NATIONAL CANCER INSTITUTE (1979) Bioassay of 2,4,6-trichlorophenol for possible carcinogenicity. *Tech. Rep. Series* **155**. DHEW Publ. No. (NIH) 79-1711.

NIOSH (1978) *Current Intelligence Bulletin* 20. Washington: Natl Inst. Occup. Safety & Health.

OLIN, G. R. & AHLBOM, A. (1980) The cancer mortality among Swedish chemists graduated during three decades: A comparison with the general population and with a cohort of architects. *Environ. Res.*, **22**, 154.

SEILER, J. P. (1978) The genetic toxicology of phenoxy acids other than 2,4,5-T. *Mutat. Res.*, **55**, 197.

SEILER, J. P. (1979) Phenoxyacids as inhibitors of testicular DNA synthesis in male mice. *Bull. Environ. Contam. Toxicol.*, **21**, 89.

VAINIO, H., PAAKKONEN, R., RONNHOLM, K., RAUNIO, V. & PELKONEN, O. (1976) A study of the mutagenic activity of styrene and styrene oxide. *Scand. J. Work Environ. Hlth*, **3**, 147.

VIANNA, N. J. & POLAN, A. (1979) Lymphomas and occupational benzene exposure. *Lancet*, i, 1394.

VIGLIANI, E. C. & FORNI, A. (1976) Benzene and leukaemia. *Environ. Res.*, **11**, 122.

VOS, J. G., MOORE, J. A. & ZINKL, J. G. (1973) Effect of 2,3,7,8-tetrachlorodibenzo-p-dioxin on the immune system of laboratory animals. *Environ. Hlth Perspect.*, **5**, 149.

UNSCEAR (1977) Sources and effect of ionizing radiation. New York: United Nations. p. 370.